# Against the Grain: Consumer’s Purchase Habits and Satisfaction with Gluten-Free Product Offerings in European Food Retail

**DOI:** 10.3390/foods13193152

**Published:** 2024-10-02

**Authors:** David Dean, Meike Rombach, Frank Vriesekoop, Philippe Mongondry, Hoa Le Viet, Sirasit Laophetsakunchai, Beatriz Urbano, Teresa Briz, Vilma Xhakollari, Güler Atasoy, Mahir Turhan, Stavroula Chrysostomou, Elena Hadjimbei, Hussein Hassan, Maya Bassil, Sanna Arnala, Dominika Głąbska, Dominika Guzek, Sophie van den Berg, Lilian Ossel, Amalia Scannell, Puja Rauniyar, Eirini Bathrellou, Meropi Kontogianni, Wim de Koning

**Affiliations:** 1 Department of Agribusiness and Markets, Lincoln University, Lincoln 7647, New Zealand; david.dean@lincoln.ac.nz (D.D.); meike.rombach@lincoln.ac.nz (M.R.); wim.dekoning@lincoln.ac.nz (W.d.K.); 2 Harper Food Innovation, Harper Adams University, Newport TF10 8NB, UK; leviethoa1998@gmail.com (H.L.V.); sirasit.l@hotmail.com (S.L.); 3 The National Institute of Origin and Quality, 49007 Angers, France; p.mongondry@inao.gouv.fr; 4 Department of Food, Technology & Bioresource Science, Groupe ESA, 49007 Angers, France; 5 Department of Agricultural and Forrest Engineering, University of Valladolid, 47002 Valladolid, Spain; beatriz.urbano@uva.es; 6 CEIGRAM, Universidad Politécnica de Madrid, 28040 Madrid, Spain; teresa.briz@upm.es; 7 School of Agriculture, Policy and Development, University of Reading, Reading RG6 6UR, UK; v.xhakollari@reading.ac.uk; 8 Department of Agricultural and Food Sciences, University of Bologna, 40126 Bologna, Italy; 9 Department of Food Engineering, University of Mersin, 33110 Mersin, Türkiye; gleratasoy@gmail.com (G.A.); mahirturhan@gmail.com (M.T.); 10 Department of Life Sciences, European University Cyprus, 2404 Nicosia, Cyprus; s.chrysostomou@euc.ac.cy (S.C.); e.hadjimbei@euc.ac.cy (E.H.); 11 School of Arts and Sciences, Lebanese American University, Beirut 03797751, Lebanon; hussein.hassan@lau.edu.lb; 12 Human Nutrition Department, Qatar University, Doha P.O. Box 2713, Qatar; bassil.maya@qu.edu.qa; 13 Keliakialiitto (Finnish Coeliac Society), 3310 Tampere, Finland; sanna.arnala@keliakialiitto.fi; 14 Institute of Human Nutrition Sciences, Warsaw University of Life Sciences, 02-787 Warsaw, Poland; dominika_glabska@sggw.edu.pl (D.G.); dominika_guzek@sggw.edu.pl (D.G.); 15 Independent Researcher, 10976 Berlin, Germany; 16 Independent Researcher, 3522TD Utrecht, The Netherlands; 17 Institute of Food and Health, University College Dublin, D04 V1W8 Dublin, Ireland; amalia.scannell@ucd.ie (A.S.); prauniyar@tirlan.ie (P.R.); 18 Department of Nutrition and Dietetics, Harokopio University, 17676 Athens, Greece; ebathrellou@hua.gr (E.B.); mkont@hua.gr (M.K.)

**Keywords:** gluten-free food products, consumer types, purchase habits, satisfaction, dissatisfaction, European retail

## Abstract

Across the world and within Europe, a growing number of consumers are choosing to buy gluten-free products. Motivations for a gluten-free diet and the consequences of consuming gluten are varied, from a medical necessity for those diagnosed with celiac disease to a range of health complications and discomfort for those who are gluten-intolerant. In this research, 7296 gluten-free consumers across 13 European countries responded to an online survey on the 33 types of gluten-free products purchased, how frequently they purchased them, their satisfaction with gluten-free quality and availability, the problems they have experienced, and the strategies they have employed to cope with these problems. The investigation examines whether and how these consumer attitudes and behaviors differ between those diagnosed with celiac disease, those who are gluten-intolerant, and those who are caregivers for others with a gluten-free diet. The results show that significant differences existed for all these habits and issues across the three gluten-free consumer groups. Specifically, caregivers purchased most of the gluten-free product types more frequently than the other two groups, experienced more availability problems, and were more likely to shop at multiple stores or make their own gluten-free products. Celiac-diagnosed consumers tended to buy gluten-free products more frequently than those who are gluten-intolerant, and they tended to be the most satisfied with the quality and range of gluten-free offerings. Despite purchasing frequency differences between the groups, the results suggest a similar hierarchy of gluten-free products that could provide the foundation for a European gluten-free food basket.

## 1. Introduction

Bread, pasta, breakfast cereals, crackers, and cookies comprising wheat, rye, and barley are stable food items in several European countries [1,2]. However, in the past two decades, these staples have been replaced by gluten-free options in many households [3,4,5,6]. The European market for gluten-free food products will be worth USD 3.62 billion (EUR 3.28 billion) in 2024 and is governed by EU regulation No. 609/2013, stipulating the labelling of food ingredients. Gluten is an ingredient that requires extra attention from food producers and marketers as it can contribute to gluten-related intolerances and can affect customers with disorders such as celiac disease [7,8,9]. Reportedly, about 1% of the EU population suffers from celiac disease, but the actual percentage is assumed to be higher, as many cases remain undiagnosed or unreported [10]. Celiac disease is an autoimmune disorder where, at present, there is no treatment available, other than removing gluten from the diet [11,12]. Accomplishing this dietary adjustment requires related lifestyle changes, which affect food consumption and purchases [13,14].

Products sold in European food retail that are labelled as ‘gluten-free’ are not allowed to exceed gluten concentrations of 20 mg/kg [7,15,16]. While gluten-free food items are closely associated with health claims and beliefs, many gluten-free food items contain highly refined flour, are lower in fiber, and are high on the glycemic index. In comparison with regular food items, they are often inferior in taste and texture. To make up for this shortcoming, manufacturers may add fats, sugars, or other ingredients to their gluten-free products. These factors, and their very high price point, are said to be hindering the growth of the European gluten-free food market [17]. Another food-safety-related issue is the cross-contamination of gluten-containing ingredients throughout the production process. Therefore, food shopping with this dietary restriction can be difficult.

The extant literature discusses consumer sensory perceptions and preferences for gluten products such as bread, pasta, and pizza [18], consumer acceptance of new product formulas, and willingness to pay for gluten-free products [19], as well as health motivations and addiction to gluten-free diets for consumers that do not require gluten-free diets. However, research dedicated to the point of sale is rather scant. The identification of a basic gluten-free European food basket and purchasing frequencies, differences among consumer groups buying gluten-free food, and their levels of product satisfaction are yet to be uncovered. Understanding differences between consumers, namely, those affected by celiac disease, those choosing to eat gluten-free food, and those that are caregivers to gluten-free consumer groups, is necessary to gain a more complete picture of the European gluten-free consumer market and these specific target groups. The present study is dedicated to addressing this gap in the literature and aims to provide best practice recommendations for marketers in European food retail. This study will provide answers to the following questions: How do the three target groups differ in their gluten-free purchasing habits and how do they assess their shopping experience of gluten-free products in European food retail?

## 2. Literature Review

### 2.1. The European Gluten-Free Food Basket

Food baskets are commonly used to indicate the monthly budget required for food purchases for reference households in European countries to reach an acceptable standard of living [20]. They are grounded in cultural food habits and informed by nutritionists and dietary guidelines. The budget accounts for food purchases, food preparation, and storage equipment. The underlying assumptions for budget and product suggestions are that households consist of children and adults who are in good health and live in capital cities [20]. Given that many of the foods typically found in a food basket are not gluten-free, gluten-free consumers do not meet the requirements of the standard basket. In addition, if celiac-diagnosed, they may not meet the health criterion, and the food basket budgets do not account for the price premiums for many gluten-free products. Thus, research toward the establishment of European gluten-free food baskets is required [11,21].

The recent body of literature provides information on gluten-free food baskets. A study from Cyprus investigated the affordability of a gluten-free food basket for people with low income [22]. The study indicated that the basket is unaffordable for low-income households, as the basket accounts for 40–60% of their income. Consequently, this group in the population is likely to be exposed to food insecurity or food stress and to be at risk of compromising their long-term health if they are forced to abandon a gluten-free diet [22]. The study confirms earlier findings from Australia, which indicated the gluten-free basket was considered unaffordable for three of the four common family types in Australia. The authors warn that for people relying on public welfare [17,23], dietary compliance is more difficult and that welfare considerations should be made to assure equitable access to gluten-free food [23].

An early study from the United Kingdom was dedicated to product assortments and the availability of gluten-free food products in physical and online shops [24]. The study found that budget stores or convenience stores had a very limited assortment. This limited assortment in budget stores was confirmed in a later study, which reported a slight increase in assorted gluten-free products in budget stores [16]. In contrast, online stores had a wider product range but often at up to twice the cost [25]. Further, Hopkins and Soon (2019) remark that from a nutritional perspective, gluten-free products such as bread, crackers, and cookies were lower in protein and sugar compared to regular products [25]. Similarly, readily prepared gluten-free meals contained less salt compared to regular meals. Consistent with previous studies, the authors criticize the high price points and the lack of availability in budget stores, which disadvantage low-income groups. Estévez et al. (2024) and Jamieson et al. (2018) identify availability, nutrition, and price point as persistent barriers to complying with a gluten-free diet [26,27].

### 2.2. Consumers Assessment of Availability and Quality of Gluten-Free Food Items

Vriesekoop et al. (2020) conducted consumer research in the United Kingdom. Their work emphasizes consumer dissatisfaction with gluten-free food items. Their dissatisfaction addresses price points, sensory experiences of staple foods items such as bread, pasta, and crackers, and shelf-life [16]. Consumers indicated improvements in the taste, texture, and smell of gluten-free foods but suggested that the mouthfeel of some gluten-free breads is reminiscent of cardboard and needs to be toasted to be palatable [16,28,29]. While bread was heavily criticized, consumers appeared to be more content with pasta [16].

These results align with consumer studies in Italy, which show that the product quality of pasta appears to be satisfactory [30]. Consumers in the UK reported unhappiness with gluten-free food going bad or moldy before use-by dates. It is unclear whether this was due to inaccurate shelf-life estimations or poor climate control, storage, or stocking procedures in the supermarkets or supply chains. The study indicated high dissatisfaction with gluten-free products regarding value for money. On a positive note, consumers in the United Kingdom trust gluten-free labelling [16]. This may be attributed to clear government labelling regulations for gluten-free products. While the study emphasizes distinct groups of people consuming gluten-free food, including people diagnosed with celiac disease and caregivers of the other consumer groups, shopping and consumption differences between these groups have not been reported [16].

### 2.3. Consumer Target Groups

Gutowski et al. (2020) and Fiori et al. (2024) provide important information about the buying behaviors of different consumer target groups [15,31]. Gutowski’s work (2020) [31] discusses the inability of consumers with celiac disease to correctly choose gluten-free items based on product labelling, as labels specify unsafe ingredients in ambiguous ways, e.g., wheat derivates. The study indicates that some consumers with celiac disease incorrectly assumed that natural and artificial flavors, cornstarch, spices, and seasonings are gluten-free products, and they misidentified soy products as gluten-containing. Consumers with celiac disease must learn to identify gluten-free food items and perfect their skills and knowledge to avoid harming themselves. Sielicka-Różyńska et al.’s (2020) eye-tracking study found that at the point of sale, gluten-free consumers actively search for written cues and that graphical indicators, such as the crossed-out grain image, only provide additional information [32].

Fiori et al. (2024) [15] investigated adherence to a gluten-free diet and report that caregivers, often parents of adolescents or small children, are extra careful and known for their strict adherence to offering a gluten-free diet. Young parents seem to be the strictest and most worried group. These individuals were more frequently concerned when thinking about food and when choosing what to eat, and they were more frequently confused when going about their grocery shopping. Approximately 40% of the sample population indicated occasionally diverging from a gluten-free diet and admitted feeling guilty about it [15]. Other studies report that some consumers of gluten-free products do not follow medical advice but follow health and wellness trends or wish to reduce their weight [33,34,35,36]. These consumers appear less strict than those with celiac disease or caregivers of those with gluten-free diets.

### 2.4. Hypothesis Development

**Figure 1:**
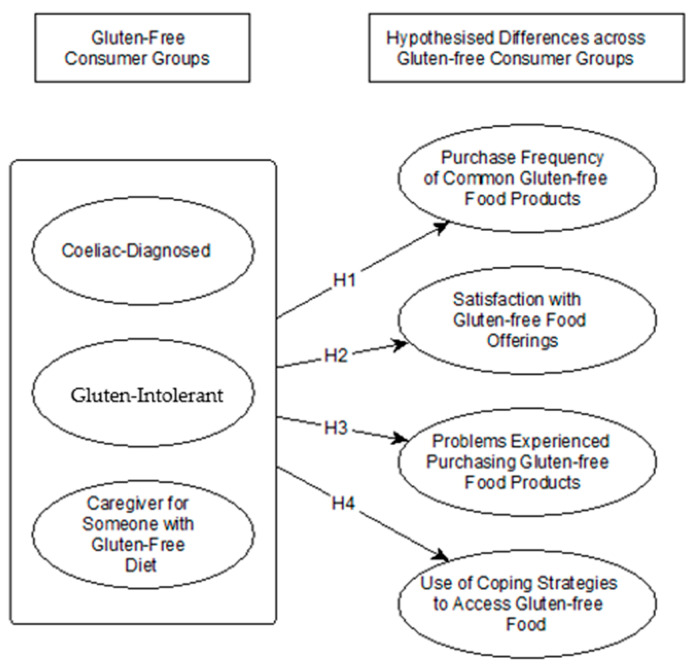
Proposed research illustration.

Building on the presented literature review, it is posited that there are attitudinal and behavioral differences between gluten-free consumer groups (see Figure 1).

The recent body of literature discusses purchasing frequencies for consumers requiring a gluten-free diet. However, to date, the understanding of purchasing frequencies has been limited to individual country contexts [16,37,38] and a general understanding of gluten-free diets and consumer demands [39]. Distinctions between celiac-diagnosed consumers, gluten-intolerant consumers, and caregivers in their purchasing frequencies have not yet been presented. Understanding the differences between these groups is important from consumer and marketer perspectives alike. Information about purchasing frequencies provides insight into demand, segmentation and targeting [40], pricing, and promotion, as well as requirements for product assortments and product innovation [41]. Purchasing frequencies may be indicators of adherence to diets in order to achieve symptom-free status and improve quality of life. On these grounds, is the following is proposed:

**Hypothesis** **1.** *Within European food retail, there are significant differences in purchasing frequencies of gluten-free food offerings between consumers following a gluten-free diet, consumers with a celiac diagnosis, and caregivers of those with gluten-free diets.*

Similarly, consumer satisfaction with gluten-free products and product assortments has been studied at the individual country level [13,16,42,43,44], but multi-country insights are rather limited [29]. There is consensus in the literature that product assortment and product availability have improved in the last decade, although recent assessments of quality and sensory experiences among gluten-free products are still mixed [13,16,29,39,44]. Examples of products that satisfy gluten-free consumers are pasta and crackers [13,16], while bread still requires the attention of food producers, as it often causes consumer dissatisfaction [6,16,29]. A distinction between consumer groups is required, as people follow a gluten-free diet for varying reasons, and their respective dependencies, product acceptance, and satisfaction with gluten-free products may vary [45]. Therefore, the following hypothesis is proposed:

**Hypothesis** **2.** *Within European food retail, there are significant differences in satisfaction with gluten-free food offerings between consumers following a gluten-free diet, consumers with a celiac diagnosis, and caregivers of those with gluten-free diets.*

Various studies have reported the challenges and problems that consumers who need to follow a gluten-free diet face when going grocery shopping [2,13,46]. These include a previously discussed limitation in product assortment or quality [16,29]. In addition, the identification/labelling of gluten-free products, cross-contamination of food, food recalls, and shopping for a nutritious diet are other challenges that are widely discussed [47,48]. The health literature emphasizes that people with gluten sensitivity from other health-related issues face the least problems, as they may be able to tolerate small amounts of gluten [31,32]. It is discussed that celiac-diagnosed consumers are more affected, as the element of care and the stronger dependency on the products intensify these outlined issues [31,32]. Coping mechanisms to mitigate these issues and distinctions among different consumer target groups are not yet widely studied [2]. Amidst this background, the following hypotheses are proposed.

**Hypothesis** **3.** *Within European food retail, there are significant differences in the problems experienced with gluten-free food offerings between consumers following a gluten-free diet, consumers with a celiac diagnosis, and caregivers of those with gluten-free diets.*

**Hypothesis** **4.** *Within European food retail, there are significant differences in the coping strategies of gluten-free food offerings between consumers following a gluten-free diet, consumers with a celiac diagnosis, and caregivers of those with gluten-free diets.*

## 3. Materials and Methods

Data from the present study stem from a multi-country investigation in Greece [GRE], the Netherlands [NLD], Belgium [BEL], France [FRA], Spain [ESP], Italy [ITA], Great Britain [GBR], Poland [POL], Turkey [TUR], Ireland [IRL], Cyprus [CYP], Finland [FIN], and Lebanon [LBN]. Ten-minute online surveys were disseminated with the assistance of the various national celiac associations in 2021. Survey participants were invited to answer questions about their consumption and purchasing habits, e.g., purchasing frequency and their assessment and reaction to their gluten-free shopping experience. To assure the comparability of purchasing frequencies, 33 products were chosen that are commonly part of European diets and that are, with some exceptions, available in each country as gluten-free alternatives. The base list of products and the survey questions were derived from Vriesekoop et al. (2020) [16]. The survey questions for the present study can be classified into three categories: sociodemographic information and gluten-free consumer group identification, as listed in Table 1, the purchasing frequency of the 33 products, as shown in Table 2, and satisfaction, problems, and coping strategies, as shown in Table 3. Because the data were merged from a series of individual country datasets, adaptations made in individual countries resulted in some issues, mainly the removal of some products due to a lack of popularity or availability (e.g., cosmetics or hair products) or the merging of food products (e.g., pasta with noodles). Overall, 20 products were collected in all 13 countries, 5 products were collected in 12 countries (flatbread, condiments, sausages, beer, cake mixes, pasta), 3 products in 11 countries (pasta, pot noodles, burgers), 1 in 10 countries (crackers), 2 in 9 countries (noodles, meal kits), and 2 in 6 countries (cosmetics, hair products).

The purposive sampling approach underpinning this study is deemed appropriate, despite being a non-probability sampling approach. A purposive sampling approach requires the researcher to determine the characteristics of the survey participants that are necessary for inclusion in the sample. In this case, the requirements were as follows: responsibility for the household food shopping, and either being sensitive to gluten, diagnosed with celiac disease, or being a caregiver for children or elderly persons who require a gluten-free diet. Recruiting was performed through national celiac associations that allow immediate access to these specific consumer groups. Recruiting through the celiac associations was critical to recruiting suitable respondents and securing reliable data, especially when considering the alternative, targeting gluten-free consumers via opt-in panel providers or crowd-sourcing platforms.

The research instrument was initially developed in English language and subsequently translated into various European languages. To assure translation accuracy and cultural appropriateness, translations were facilitated by individuals who are co-authors of this work, are native speakers, and use English as their professional language. In health and dietary studies, translation accuracy has become increasingly important in the last two decades [49,50]. This study received ethical approval through Harper Adams University, United Kingdom, with the identification number 0439-202106-STAFF.

After data cleaning, the sample consisted of 7296 European consumer respondents. Cleaning involved the deletion of responses which were incomplete or had not been carefully completed. Online surveys are often subject to speeding behavior, where survey participants complete a survey much faster than the average completion time. The data were analyzed via the software package SPSS 29, using descriptive statistics to describe the European consumer sample. Analysis of variance (one-way ANOVA) and the Games–Howell post hoc tests were used to identify differences among the three consumer target groups. One-way ANOVA is a parametric test specifically used to determine if there are statistically significant differences between the means of two or more independent groups (in this case, the three consumer groups). If significant differences are found (using F statistics at *p* < 0.05), this provides the justification necessary to use post hoc analyses to identify whether the responses of specific groups are statistically significantly different from each other. The choice of the specific post hoc test is based on group sizes and parametric assumptions [51]. One such assumption is equality of variance, tested using the Levene statistic, where if significant (*p* < 0.05), it indicates that post hoc tests should be limited to those that do not assume equality of variance, such as the Games–Howell post hoc test, which is appropriate for uneven group sizes and is not limited to small samples [39,40]. These post hoc tests provide confidence intervals for group mean differences and indicate whether each pairwise comparison is statistically significant (*p* < 0.05).

## 4. Results

The overall sample can be described as predominantly female and consisted of 15.4% men and 84.4% women. Only 0.3% of the sample identified as a different gender concept or preferred not to reveal their gender identity. Approximately 64.6% had been diagnosed with celiac disease, 14.2% were affected by gluten intolerance, and 21.2% indicated being a caregiver of someone with a gluten-free diet. In terms of age, the sample was reasonably well balanced, with the exception of the age groups 65–84 and 85+, which amounted to a total of 8.3%. Mid-age groups, namely 35–44 and 45–54 years old, made up much of the sample, with 47.3%. The individual information for each country can be obtained from Table 1.

To test Hypothesis 1, gluten-free consumers were presented with 33 gluten-free product types and asked how frequently they purchased the product type, with the response options of 0 = never, 1 = seldom, 2= sometimes, and 3 = often. Table 2 shows that except for “ready-meals”, all the gluten-free product categories had significant one-way ANOVAs (*p* < 0.05), indicating differences in purchasing frequency across the three gluten-free consumer groups. This was sufficient evidence to claim support for Hypothesis 1. Post hoc tests were performed to establish significant differences between the groups. Caregivers generally reported higher purchasing frequencies compared with the other groups. Specifically, caregivers reported higher purchasing frequencies than both the celiac-diagnosed and gluten-intolerant groups for 19 of the 33 product types and higher than only one of the other groups for 9 product types. The group with the second-highest purchasing frequency was those with a celiac diagnosis, reporting higher purchasing frequencies than both the other groups for 3 product types and higher than only one other group for 18 product types. The gluten-intolerant group reported higher purchasing frequencies than both the other groups for only one product type and higher purchasing frequency for only one other group for five product types.

Table 2 also presents the product type results sorted by the highest overall mean purchasing frequency. The rankings of product types for the consumer groups are also reported to highlight any notable deviations (bolded), defined as a deviation of five or more ranking positions from the overall ranking. Overall, and for all the consumer groups, the most frequently purchased gluten-free product types were pasta, bread, flour, and biscuits/cookies, and the least purchased product types were couscous, meal kits, ready-meals, and cake mixes. The celiac group had no notable deviations from the overall ranking, but this consistency can be explained by the fact that it was the largest of the three groups in the sample. The caregivers also had a consistent ranking, with only two notable deviations (crackers and pot noodles). The gluten-intolerant group diverged the most with eight notable deviations. Finally, a mean rank deviation score was calculated as the average absolute value of the ranking deviations and is reported in Table 2. The celiac-diagnosed group had a mean rank deviation of less than 1 rank position (0.67), the caregivers deviated more with 1.82 rank positions, and the gluten-intolerant group deviated the most, deviating 2.67 rank positions.

Hypothesis 2 was tested by examining seven satisfaction items measured on five-point Likert scales. One-way ANOVAs showed that the groups reported significantly different satisfaction ratings, supporting Hypothesis 2 (see Table 3). Post hoc analyses showed that the celiac-diagnosed group reported higher levels of satisfaction than both the gluten-intolerant and carer consumer groups for three of the items and higher than just the carer group for three items. Perhaps this is an indication that those with celiac diagnoses are simply more appreciative of having gluten-free options to choose from. Like in Table 2, the satisfaction scores were sorted by the highest overall scores, showing that “trust in the labels” was positive (3.720 or ~ agree) and “enjoyment of the gluten-free offerings” and “satisfaction with the quality and range of gluten-free offerings” was neutral (2.730 to 3.160 or ~ neutral). Responses for “gluten-free offerings as good as non-gluten-free offerings” and “the ‘Free-from’ aisles are well stocked” were neutral to negative (2.380–2.470 or ~ neutral/disagree), and “gluten-free offerings are good value” was negative (2.150 or ~ disagree). This suggests that the quality and range of gluten-free offerings are good, but the range could be better stocked and are often overpriced.

Hypothesis 3 was tested by examining two items asking whether the gluten-free consumers had experienced problems with gluten-free offerings. One-way ANOVAs were significant for both items, providing support for Hypothesis 3. Post hoc analyses revealed that caregivers had experienced more availability problems than the other groups. Overall, the results were not positive, with 79% of the gluten-free consumers experiencing problems with availability and 66% experiencing problems with the quality of gluten-free offerings.

Hypothesis 4 was tested by examining differences across the groups in using two coping strategies for issues in the quality or availability of gluten-free offerings. One-way ANOVAs were significant for both strategies, supporting Hypothesis 4. Post hoc analyses indicated that caregivers were more likely than the other groups to shop at several stores to find all the gluten-free products they needed. The caregivers were also more likely to make their own gluten-free products than the celiac-diagnosed consumers.

## 5. Discussion

The celiac-diagnosed, caregivers of the gluten-free, and the gluten-intolerant are related but distinct segments of gluten-free consumers. Caregivers seemed to have the highest purchasing frequency for most of the gluten-free offerings throughout the most popular to the least popular product groups. They were the least satisfied with the gluten-free product offerings, quality, and availability. They experienced more problems with availability, and they engaged in more coping strategies to obtain the products they need.

There are several possible explanations for these findings. First, caregivers are often parents, family, or professional caregivers, and since their charges are the ones who will experience the uncomfortable-to-dangerous consequences of gluten contamination, they are likely to be more vigilant providers of gluten-free food. Also, unless they and the rest of the household follow a gluten-free diet, the carers are more likely to rely on packaged foods that are known to be gluten-free for their charges. Food that is clearly labelled and can be kept separate from non-gluten-free food is much easier to manage in such a situation, so this could explain why prepared gluten-free foods such as breakfast cereals, condiments, pizza, sausages, and sweets are more common on a caregiver’s shopping list. These findings complement medical and dietary studies. Following Caetano-Silva et al. (2024), a gluten-free diet requires eating competence from the affected person or their caregivers [52]. A gluten-free diet requires strict compliance, knowledge about food products and their ingredients, meal planning, food storage, and meticulous attention to product choices. Caregivers of children or the elderly must have the highest degree of food competence, which involves shopping, food preparation, and consumption [52]. Bariyah et al. (2024) also indicate that caregivers have moderate-to-high knowledge about gluten-free diets, but they experience problems when putting this knowledge into practice [53]. The study outlines accessing food ingredients, costs, and quality as major issues to strictly following a gluten-free diet. Hameed and Sondhi (2023) acknowledge the critical role of the caregiver [54]; while the findings of the present study echo their findings related to food competence, they further emphasize that attitudes towards the disease, income, education, and influence of other parties in the household play an important role on a caregiver buying and consumption behavior. Non-acceptance of the disease, pressure, switching behavior, and distrust towards products and out-of-home eating are addressed in the study.

Adult celiac-diagnosed consumers tended to purchase most of their gluten-free products less frequently than caregivers but more than the gluten-intolerant group. Because of the medical consequences, it is unlikely that this group will eat any non-gluten-free food, so they have likely changed their food preferences, meaning that they do not often need to buy some gluten-free versions of typical products. While they may have developed the culinary skills to make their own gluten-free options, they reported using this coping strategy less than the caregiver group. Other explanations could include having more out-of-home meals or eating less processed foods like pizza and sausages. At present, there is no information on the purchasing frequency of celiac-diagnosed consumers in comparison with caregiver or gluten-intolerant consumers in multiple European countries. However, it is known that shopping and consumption habits, including purchasing frequency, contribute to food competency and success in adherence to gluten-free diets [15,55].

Finally, the gluten-intolerant group was distinct from the other gluten-free consumer groups, in that most gluten-free products were purchased less frequently, with the notable exception of porridge/oats. This group seemed to be less satisfied than the celiac-diagnosed group, experienced fewer problems than caregivers, and were less likely to shop at several stores to obtain gluten-free products. Their shopping behavior was consistent with someone who feels better when they avoid eating gluten but does not face life-threatening consequences when they do not. Perhaps their higher frequency of buying gluten-free porridge/oats is the best evidence of this. For many gluten-intolerant consumers, the level of possible cross-contamination of gluten from other grains may not be a problem, but for celiac-diagnosed consumers, it could be. Some studies outline that gluten-free food is consumed because consumers believe that gluten-free products are healthier, help improve other medical conditions, are helpful to reducing weight, and can mitigate acne [6,56]. Moreover, consumers not required to follow a gluten-free diet may still do so for psychological or well-being reasons [6]. Moreover, other non-celiac, but gluten-sensitive, disorders may allow for some variation in the gluten-free diet. Those diets may allow for certain non-wheat cereals that contain gluten analogues or allow for limited quantities of gluten resulting from cross-contamination [57].

Another finding of this study is that there seems to be a hierarchy of gluten-free products for most European gluten-free consumers. Coupled with concerns about large price premiums for gluten-free products, knowing the most common products could be beneficial for establishing gluten-free food baskets and ultimately gluten-free budgets. While pasta, bread, flour, and biscuits/cookies top the purchasing frequency, it could be argued that any gluten-free product with a purchasing frequency score of ≧1.5 (midpoint between “seldom” and “sometimes” alternatives) should be considered for such a basket. For caregivers, this list would include 15 products, for the celiac-diagnosed, 9 products, and for the gluten-intolerant, 8 products.

## 6. Conclusions

The present study fills an important gap in the literature, with its focus on the comparison of three consumer target groups and with respondents from multiple European countries. Studies with consumer data from multiple countries are rare, but they are increasingly important as gluten sensitivity and celiac diagnoses continue to be an increasing health issue across the European market. The knowledge gained about the purchasing habits, satisfaction, and coping mechanisms of gluten-free consumers and caregivers complements research in the medical, health, and dietary fields. Scholars in these disciplines have frequently called for this information, as strict dietary adherence is the only way for consumers requiring a gluten-free diet to lead healthy lives. Understanding purchasing habits, satisfaction, and coping mechanisms can be important factors contributing to disease management. While product availability and consumer satisfaction/dissatisfaction are receiving wider attention in recent times, coping strategies have been underexplored. Coping strategies are an important predictor of food knowledge and eating competency, as the coping mechanisms result from the absence or inconsistent availability of desired gluten-free items. The absence of desired products triggers a search for alternative offerings and consideration of the opportunity cost to undertake additional shopping in other retailers. This requires a wider understanding of retail offerings and sufficient food knowledge to consider alternative food options. Deepening the knowledge of caregivers, their diets, and the impact on their dependents is a promising avenue for future studies. The paradox of marketing to caregivers is likely an interesting lens.

As such, the current work is of interest to both food and health marketers. Marketing campaigns should address the development of food competence and work with influencers to support the educational efforts of medical practitioners and celiac associations. Marketing campaigns should be grounded in lifestyle marketing, as following a gluten-free diet is a major lifestyle adjustment to the food purchasing and eating habits of the affected person, their immediate family, and their social circles. Lifestyle marketing allows for the positioning of gluten-free food products or food-related and dietary services to possess the needs, desires, and aesthetics that the target audience identifies with.

While this study shows that product assortments have been improved, quality, value for money, and availability were still seen as barriers for all the gluten-free consumer target groups. Food retailers who are willing to mitigate these issues may have a unique advantage in distinguishing themselves from competitors. Given that gluten-free diets are becoming a necessity for an increasing number of people in Europe, price points and food basket recommendations should account for this. This issue can lead to food inequality and requires a wider discussion among policymakers, health professionals, and the food industry.

Future research should focus on gluten-free food basket choice experiments following Caputo and Lusk (2022) [58]. Such work may help inform food-related food security and welfare policies. The purchasing frequencies from the current study coupled with a detailed analysis of prices for the most purchased gluten-free products could provide an excellent foundation for a European gluten-free food basket and associated household budget. Furthermore, further work could focus on generational cohorts and gluten-free consumption, understanding the perspectives of caregivers and young consumers in the context of brand loyalty or switching behavior for bread, pasta, cereals, and other selected products. While the high price point of products suggests switching behavior to be likely, the sensory properties of specific products and brands may suggest loyalty.

## Figures and Tables

**Table 1:** Sample description.

Country	GRE	NLD	BEL	FRA	ESP	ITA	GBR	POL	TUR	IRL	CYP	FIN	LBN	Overall
Sample Size	453	615	390	343	1033	903	198	1036	406	930	238	488	263	7296
Celiac/GI/Carer														
Celiac Diagnosis	43.3%	68.9%	45.6%	67.6%	68.6%	73.2%	78.3%	62.1%	62.6%	72.9%	25.2%	90.4%	30.4%	64.6%
Gluten Intolerance	26.5%	12.4%	33.1%	15.5%	9.3%	3.8%	14.1%	6.8%	18.2%	9.2%	47.1%	5.5%	51.0%	14.2%
Caregiver for CD/GI person	30.2%	18.7%	21.3%	16.9%	22.1%	23.0%	7.6%	31.2%	19.2%	17.8%	27.7%	4.1%	18.6%	21.2%
Gender														
Male	15.0%	16.1%	9.2%	13.7%	14.9%	13.8%	22.7%	9.2%	17.2%	18.2%	22.3%	20.1%	23.2%	15.4%
Female	85.0%	83.6%	90.3%	86.3%	85.0%	85.8%	76.8%	90.5%	82.5%	81.6%	77.7%	79.1%	76.8%	84.4%
Prefer Other	0.0%	0.3%	0.5%	0.0%	0.1%	0.3%	0.5%	0.3%	0.2%	0.2%	0.0%	0.8%	0.0%	0.3%
Age Groups														
<18	1.8%	4.1%	3.6%	6.1%	6.4%	4.8%	0.0%	5.1%	14.3%	4.4%	1.3%	0.8%	4.6%	4.8%
18–24	10.4%	6.2%	6.2%	7.9%	9.2%	7.5%	6.6%	5.9%	16.3%	3.1%	10.9%	1.0%	27.8%	7.8%
25–34	27.6%	15.1%	21.3%	17.8%	22.0%	24.9%	6.6%	29.9%	34.0%	9.2%	23.1%	6.1%	28.1%	20.8%
35–44	30.5%	18.7%	32.8%	31.2%	27.5%	31.0%	9.1%	40.6%	26.1%	19.4%	36.6%	15.4%	21.3%	27.3%
45–54	20.5%	19.8%	20.8%	17.8%	25.8%	21.0%	18.2%	14.8%	7.4%	25.6%	21.0%	20.5%	13.7%	20.0%
55–64	7.7%	15.0%	12.1%	12.5%	7.5%	8.9%	21.2%	3.2%	1.2%	21.6%	5.9%	26.6%	1.9%	11.0%
65–74	1.5%	13.7%	2.8%	4.7%	1.5%	1.7%	27.8%	0.4%	0.7%	12.8%	1.3%	23.4%	1.1%	6.2%
75+	0.0%	7.5%	0.5%	2.0%	0.2%	0.2%	10.6%	0.1%	0.0%	3.9%	0.0%	6.1%	1.5%	2.1%

**Table 2:** Gluten-free product type purchase frequency by consumer group.

GF Product Purchase Frequency	Celiac Diagnosis			Gluten Intolerance			Caregiver for CD/GI Person			Overall			One-Way ANOVA	
	Mean	St Dev	Rank	Mean	St Dev	Rank	Mean	St Dev	Rank	Mean	St Dev	Rank	F Stat	*p*-Value
Pasta	2.448 _GI_	0.810	1	2.189	1.015	1	2.69 _CD,GI_	0.678	1	2.465	0.828	1	99.726	<0.001
Bread	2.357 _GI_	0.922	2	1.935	1.072	3	2.42 _GI_	0.909	3	2.310	0.955	2	98.384	<0.001
Flour	2.185 _GI_	0.944	3	1.948	1.065	2	2.444 _CD,GI_	0.821	2	2.206	0.949	3	90.056	<0.001
Biscuits/Cookies	1.900 _GI_	0.926	4	1.650	1.050	4	2.291 _CD,GI_	0.834	4	1.947	0.947	4	165.731	<0.001
Chocolate	1.869 _GI_	0.968	5	1.621	1.115	5	2.078 _CD,GI_	0.916	6	1.878	0.989	5	67.847	<0.001
Crisps	1.750 _GI_	1.016	6	1.436	1.126	9	2.08 _CD,GI_	0.960	5	1.775	1.039	6	126.985	<0.001
Ice Cream	1.598 _GI_	0.948	8	1.197	1.061	**14**	1.955 _CD,GI_	0.930	7	1.617	0.986	7	194.692	<0.001
Crackers	1.603	1.073	7	1.518	1.129	7	1.68 _GI_	1.027	**13**	1.607	1.072	8	5.035	0.007
Raising Agents	1.561 _GI_	1.077	9	1.261	1.133	11	1.844 _CD,GI_	1.052	8	1.578	1.094	9	91.883	<0.001
Breakfast Cereal	1.478 _GI_	1.166	11	1.327	1.167	10	1.777 _CD,GI_	1.156	9	1.520	1.173	10	54.734	<0.001
Rice Cakes	1.432	1.127	13	1.534 _CD_	1.185	**6**	1.691 _CD,GI_	1.141	12	1.501	1.143	11	30.437	<0.001
Condiments	1.496 _GI_	1.077	10	1.198	1.147	13	1.607 _CD,GI_	1.122	15	1.481	1.102	12	40.312	<0.001
Pizza	1.449 _GI_	1.038	12	1.094	1.072	17	1.741 _CD,GI_	1.050	10	1.460	1.062	13	119.632	<0.001
Sausages	1.427 _GI_	1.098	14	0.894	1.084	**23**	1.683 _CD,GI_	1.119	14	1.401	1.125	14	144.186	<0.001
Sweets	1.389 _GI_	0.999	15	0.971	1.021	19	1.702 _CD,GI_	0.987	11	1.396	1.022	15	166.351	<0.001
Porridge/Oats	1.307 _C_	1.242	16	1.509 _CD,C_	1.256	**8**	1.207	1.182	20	1.315	1.234	16	18.829	<0.001
Noodles	1.149	1.072	19	1.158	1.108	16	1.392 _CD,GI_	1.136	16	1.200	1.093	17	23.352	<0.001
Stock Cubes	1.219 _GI_	1.123	17	0.966	1.115	20	1.269 _GI_	1.193	19	1.194	1.141	18	25.312	<0.001
Hair Products	1.217 _GI,C_	1.168	18	0.916	1.124	21	1.094 _GI_	1.155	22	1.151	1.164	19	14.043	<0.001
Flat Bread	1.071	1.001	22	1.238 _CD_	1.125	**12**	1.323 _CD_	1.074	17	1.149	1.040	20	36.424	<0.001
Cereal Snack Bars	1.075	1.045	20	1.178 _CD_	1.119	**15**	1.321 _CD,GI_	1.113	18	1.141	1.075	21	31.344	<0.001
Cakes	1.006	0.874	23	0.994	0.977	18	1.19 _CD,GI_	0.953	21	1.044	0.909	22	25.497	<0.001
Cosmetics	1.073 _GI_	1.133	21	0.784	1.032	**26**	0.984 _GI_	1.101	26	1.016	1.117	23	13.069	<0.001
Pastry	0.917	0.905	24	0.912	0.973	22	1.062 _CD,GI_	1.000	23	0.947	0.938	24	14.901	<0.001
Sauces	0.903	1.057	25	0.824	1.031	24	1.03 _CD,GI_	1.133	24	0.918	1.072	25	12.978	<0.001
Soup	0.874 _GI,C_	1.029	26	0.709	1.009	27	0.779	1.034	29	0.830	1.029	26	13.362	<0.001
Burgers	0.791 _GI_	1.011	28	0.664	0.974	29	0.908 _CD,GI_	1.108	27	0.798	1.030	27	14.047	<0.001
Beer	0.848 _GI,C_	1.036	27	0.698	0.995	28	0.522	0.889	33	0.760	1.010	28	61.726	<0.001
Cake Mixes	0.676	0.891	30	0.629	0.912	31	1.034 _CD_	1.018	25	0.746	0.934	29	93.134	<0.001
Ready Meals	0.714 _GI_	0.922	29	0.638	0.929	30	0.713	0.934	30	0.703	0.926	30	2.951	0.052
Pot Noodles	0.550	0.963	31	0.810 _CD_	1.137	**25**	0.858 _CD_	1.153	28	0.648	1.038	31	58.589	<0.001
Meal Kits	0.539	0.810	32	0.471	0.810	33	0.632 _CD,GI_	0.895	31	0.548	0.827	32	7.856	<0.001
Couscous	0.390	0.723	33	0.539 _CD_	0.874	32	0.577 _CD_	0.859	32	0.451	0.781	33	41.217	<0.001
Mean Frequency Score	1.281	1.006		1.134	1.059		1.442	1.019		1.294	1.026			
Mean Rank Deviation			0.67			2.67			1.82			0.00		

_CD_ = higher agreement than the celiac-diagnosed group (Games–Howell test <0.05); _GI_ = higher agreement than the gluten-intolerant group (Games–Howell test <0.05); _C_ = higher agreement than the caregiver group (Games–Howell test <0.05). **Bold** = change in rank ≧ 5 positions compared to overall sample.

**Table 3:** Satisfaction, problems, and coping strategies by consumer group.

Question/Item	Celiac Diagnosis		Gluten Intolerance		Caregiver for CD/GI Person		Overall		One-Way ANOVA	
	Mean	St Dev	Mean	St Dev	Mean	St Dev	Mean	St Dev	F Stat	*p*-Value
Satisfaction with Gluten-Free (GF) Offerings (1 = Strongly Disagree to 5 = Strongly Agree)										
I trust the labels of GF offerings	3.772 _GI,C_	1.183	3.537	1.245	3.69 _GI_	1.168	3.720	1.192	17.536	<0.001
I enjoy the GF offerings	3.192 _C_	1.070	3.138	1.134	3.070	1.032	3.160	1.072	7.633	<0.001
I’m satisfied with the quality of GF offerings	2.898 _GI,C_	1.113	2.776	1.183	2.810	1.060	2.860	1.114	7.004	<0.001
I’m satisfied with the range of GF offerings	2.803 _GI,C_	1.156	2.591	1.205	2.540	1.115	2.720	1.161	37.991	<0.001
GF offerings are as good as non-GF offerings	2.443	1.191	2.519	1.258	2.520	1.198	2.470	1.203	3.626	0.027
The “Free-from” aisles are well stocked	2.428 _C_	1.115	2.385 _C_	1.134	2.210	1.087	2.380	1.115	22.072	<0.001
The GF offerings represent good value	2.178 _C_	1.165	2.154	1.143	2.080	1.137	2.150	1.157	4.227	0.015
Problems with Gluten-Free Offerings (No = 1 to Yes = 2)										
I have experienced availability problems	1.784	0.411	1.784	0.411	1.834 _CD,GI_	0.372	1.790	0.404	9.202	<0.001
I have experienced quality problems	1.693	0.461	1.564	0.496	1.640	0.480	1.660	0.472	34.316	<0.001
Gluten-Free Coping Strategies (1 = Strongly Disagree to 5 = Strongly Agree)										
I shop at several stores to get everything	3.972 _GI_	1.289	3.853	1.303	4.24 _CD,GI_	1.197	4.010	1.278	34.63	<0.001
I make my own gluten-free products	3.436	1.388	3.483	1.437	3.59 _CD_	1.295	3.480	1.377	7.285	<0.001

_CD_ = higher agreement than the celiac-diagnosed group (Games–Howell test <0.05); _GI_ = higher agreement than the gluten-intolerant group (Games–Howell test <0.05); _C_ = higher agreement than the caregiver group (Games–Howell test <0.05).

## Data Availability

The data presented in this study are available on request from the corresponding author.
